# Antifungal and Antibacterial Metabolites from a French Poplar Type Propolis

**DOI:** 10.1155/2015/319240

**Published:** 2015-03-22

**Authors:** Séverine Boisard, Anne-Marie Le Ray, Anne Landreau, Marie Kempf, Viviane Cassisa, Catherine Flurin, Pascal Richomme

**Affiliations:** ^1^EA 921 SONAS/SFR 4207 QUASAV, Université d'Angers, 16 boulevard Daviers, 49045 Angers Cedex 01, France; ^2^Laboratoire de Bactériologie-Hygiène, Centre Hospitalier Universitaire, 4 rue Larrey, 49933 Angers Cedex 09, France; ^3^Groupe d'Etude des Interactions Hôte Pathogène (GEIHP), Université d'Angers, 4 rue Larrey, 49933 Angers Cedex, France; ^4^Ballot-Flurin Apiculteurs-Abeilles Santé, 75 place Lagardère, 65700 Maubourguet, France

## Abstract

During this study, the *in vitro* antifungal and antibacterial activities of different extracts (aqueous and organic) obtained from a French propolis batch were evaluated. Antifungal activity was evaluated by broth microdilution on three pathogenic strains: *Candida albicans, C. glabrata*, and *Aspergillus fumigatus*. Antibacterial activity was assayed using agar dilution method on 36 Gram-negative and Gram-positive strains including *Staphylococcus aureus*. Organic extracts showed a significant antifungal activity against *C. albicans* and *C. glabrata* (MIC_80_ between 16 and 31 *µ*g/mL) but only a weak activity towards *A. fumigatus* (MIC_80_ = 250 *µ*g/mL). DCM based extracts exhibited a selective Gram-positive antibacterial activity, especially against *S. aureus* (SA) and several of its methicillin-resistant (MRSA) and methicillin-susceptible (MSSA) strains (MIC_100_ 30–97 *µ*g/mL). A new and active derivative of catechin was also identified whereas a synergistic antimicrobial effect was noticed during this study.

## 1. Introduction

Propolis is a resinous natural substance collected by honeybees from buds and exudates of various trees and plants, mixed with beeswax and salivary enzymes. Bees generally use it as a sealer, to smooth out the internal walls of the hive, as well as a protective barrier against intruders. Propolis has been used in folk medicine since ancient times due to its pharmacological potential associated with antioxidant [1–3], antifungal [4, 5], antibacterial [6–8], and anti-inflammatory [9] properties.

Propolis is generally composed of 50% of resin and balm (including polyphenolic compounds), 30% of wax and fatty acids, 10% of essential oils, 5% of pollen, and 5% of various organic and inorganic compounds. However, the composition of propolis deeply depends on the vegetation at the site of collection [10]. Indeed, propolis from temperate climatic zones, like in Europe, North America, or nontropical regions of Asia, mainly originates from the bud exudates of* Populus* species (*Salicaceae*) and consequently is rich in flavonoids and phenolic acids and their esters [11]; however tropical propolis, originating from regions where neither poplars nor birches grow, is rich in prenylated derivatives of* p*-coumaric acids, benzophenones, or terpenoids [12, 13].

The antifungal, antibacterial properties and chemical composition of propolis from many countries all over the world have been widely studied [6, 8, 14–20] but few reports were already given for European propolis [21, 22]. In 1990, Grange and Davey [23] highlighted for the first time the bactericidal activity of a French propolis against Gram-positive strains whereas later on, in 2000, Hegazi et al. [22] could associate this antibacterial activity with the presence of benzyl caffeate, pinocembrin, and* p*-coumaric acid.

During a previous study, we have evaluated the antioxidant and anti-AGEs activities of different solvents extracts [water; 95% EtOH; 70% EtOH; MeOH; dichloromethane (DCM) and DCM/MeOH/H_2_O (31/19/4)] obtained from a French propolis batch and identified their active constituents [24]. Here we have investigated the* in vitro* antifungal and antibacterial activities of these extracts. The antifungal activity was studied on three fungal strains (two yeasts,* Candida albicans* and* C. glabrata,* and one filamentous fungus,* Aspergillus fumigatus*). 36 strains of Gram-positive (including* Staphylococcus aureus*) and Gram-negative (including* Escherichia coli*) bacteria were used for the antibacterial assays. During this study, a new secondary metabolite was isolated, namely, 8-[(E)-phenylprop-2-en-1-one]-5-methoxy-(±)-catechin.

## 2. Materials and Methods

### 2.1. Reagents and Standards

Formic acid,* p*-coumaric acid, ferulic acid, isoferulic acid, 3,4-dimethoxycinnamic acid, and prenyl caffeate were purchased from Sigma-Aldrich (L'Isle d'Abeau Chesnes, Saint-Quentin-Fallavier, France). Caffeic acid and chrysin were obtained from Acros Organics (Geel, Belgium). Galangin was purchased from Extrasynthese (Genay, France). Pinocembrin and pinobanksin-3-acetate were isolated from the DCM extract of propolis.

### 2.2. Instrumentation

Optical rotation was measured on a JASCO P-2000 polarimeter. IR spectra were recorded on a Bruker Vertex 70 spectrophotometer. NMR spectra (1D and 2D) were recorded on a Bruker Avance spectrometer at 500 MHz for ^1^H and 125 MHz for ^13^C. MS analyses were performed on an ESI/APCI Ion Trap Esquire 3000+ from Bruker. UV absorbances were obtained from a Tecan Infinite M200 microplate spectrophotometer.

### 2.3. Propolis Samples

In order to analyze a typical French batch, that is, exhibiting an average chemical composition, a mixture of samples (10 g of each), collected in apiaries originating from different regions of France, was used for this study. These samples were provided by “Ballot-Flurin Apiculteurs,” a company specialized in organic beekeeping. Indeed, even collected in the same geographical region, propolis profiles may differ between apiaries and even inside the same apiary from one hive to another one [25]. Keeping in mind any potential economic development, it then appeared more appropriate to study an industrial end-product, that is, a mixture, exhibiting an average chemical composition associated with an average antimicrobial activity, rather than a specific sample. Therefore, 24 batches of propolis collected over two years (2010 and 2011) from different places in France (cf. supporting information 1; see Supplementary Material available online at http://dx.doi.org/10.1155/2015/319240) were homogeneously mixed to undergo this study.

### 2.4. Extractions

The extraction processes have been already described [24]. Briefly, the propolis batch was homogeneously pulverized in the presence of liquid nitrogen and divided into 1 g samples. Four different extractions were then carried out on 1 g samples with water (E1), 95% EtOH (E2), 70% EtOH (E3), and MeOH (E4). Then, two extractions, preceded by a cyclohexane wax elimination, were independently performed on 1 g samples with DCM (E5) and a mixture of DCM, MeOH, and H_2_O (31/19/4) (E6). For E1, a decoction of 1 g of propolis powder was boiled in 20 mL H_2_O at 100°C for 15 min. After cooling, the solidified wax and the residue were removed by filtration, and the filtrate was evaporated to dryness. For other solvents, 1 g of propolis powder (or residue obtained from a previous extraction) was macerated in 3 × 20 mL of solvent. After stirring for 3 × 2 h at room temperature, the mixture was filtered. The filtrates were gathered and evaporated under vacuum. Extraction yields (dried extract/100 g) were as follows: E1 7%; E2 68%; E3 65%; E4 68%; E5 50%; and E6 59%.

### 2.5. Antifungal Activity

Antifungal activity was assayed on human pathogenic fungi, including two common yeasts (*Candida albicans* ATCC 66396 and* C. glabrata* LMA 90-1085) and an opportunistic mould (*Aspergillus fumigatus* CBS 11326). The strains were obtained from the Parasitology and Mycology Laboratory at the University Hospital Center of Angers, France. Microorganisms were cultivated at 37°C on yeast extract-peptone-dextrose-agar (YPDA) containing 0.5 g/L chloramphenicol for two (*C. albicans* and* C. glabrata*) or three (*A. fumigatus*) days. Tests were performed according to a procedure described by Alomar et al. [27], following the guidelines of the approved reference method of the National Committee for Clinical Laboratory Standards (NCCLS) for yeasts [28] and filamentous fungi [29]. Briefly, the yeast suspensions were prepared in RPMI-1640 culture medium and adjusted spectrophotometrically at 630 nm to reach a final concentration of* ca*. 0.5 × 10^3^ to 2.5 × 10^3^ cells/mL. The tests were performed using sterile 96 flat shaped well microtiter plates. Serial twofold sample dilutions were made in DMSO. Sample solutions were dispensed at a volume of 5 *μ*L in triplicate into the wells to obtain final concentrations from 250 to 1.95 *μ*g/mL. After 48 h at 37°C for* C. albicans* and* C. glabrata* and 72 h for* A. fumigatus*, the spectrophotometric MIC endpoint was calculated from the turbidimetric data as the lowest sample concentration causing a growth inhibition equal to or greater than 80% of the control (MIC_80_). Amphotericin B was used as a positive control.

### 2.6. Antibacterial Activity

Antibacterial activity was evaluated on 36 human pathogenic bacterial strains collected by the Laboratory of Bacteriology at the University Hospital Center of Angers, France: seven strains of* Acinetobacter baumannii* (RCH, SAN008, 12, AYE, CIP7034, 107292, and 5377), two of* Escherichia coli* (ATCC25922 and a clinical isolate), three of* Pseudomonas aeruginosa *(ATCC27853 and two clinical isolates), and 4 clinical isolates of* Enterobacter cloacae*,* E. aerogenes*,* Klebsiella oxytoca*, and* Salmonella enteritidis* (phage type 4) for Gram-negative bacteria; thirteen strains of* Staphylococcus aureus* (ATCC25923, six methicillin-susceptible clinical isolates, six methicillin-resistant clinical isolates), two clinical isolates of* S. epidermidis* (*methiS* and* methiR*), three clinical isolates of* Enterococcus faecalis* and one of* E. faecium*, and one clinical isolate of* Corynebacterium striatum* for Gram-positive bacteria. Tests were performed using the methodology described by Alomar et al. [30]. Briefly, a stock solution of each sample was prepared in triplicate at 20 mg/mL in DMSO under sterile conditions. Serial dilutions were prepared (sample concentrations: 10, 20, 30, etc., to 100 *μ*g/mL) and 0.1 mL of each dilution was added to 19.9 mL of Mueller-Hinton agar (Merck, Germany) and transferred to Petri plates. Bacterial strains (2 × 10^4^ CFU/mL) were suspended in sterile NaCl aqueous solution (0.15 M) and inoculated on the different Petri plates using the multipoint inoculator (AQS, England). After 24 h of incubation at 37°C, the minimum inhibitory concentration (MIC_100_, *μ*g/mL) against bacterial strains was defined as the lowest concentration of each sample that inhibited visible growth. A blank was made inoculating the strains on Mueller-Hinton agar without any extract or compound. Oxacillin was used to distinguish the methicillin-resistant from the susceptible staphylococcal strains.

### 2.7. HPLC-DAD and HPLC-MS Procedures

Dry extracts were dissolved in MeOH (5 mg/mL for the aqueous extract and 10 mg/mL for the organic solvents ones) and centrifuged at 13000 rpm for 10 min prior to injection (10 *μ*L) into the HPLC system. Analytical HPLC was run on a 2695 Waters separation module equipped with a diode array detector 2996 Waters. Separation was achieved on a LiChrospher column 100 RP-18 (125 × 4 mm i.d., 5 *μ*m) protected with a LiChrocart 4-4 guard cartridge (4 × 4 mm i.d.) at a flow rate of 1 mL/min. The mobile phase consisted of 0.1% formic acid in water (solvent A) and MeOH (solvent B) and the separation was performed using the linear gradient: 25–100% B in 40 min. UV detection was achieved at two wavelengths: 254 and 280 nm.

The mass analyses were performed with an ESI interface coupled to an ion trap mass analyzer in both positive and negative modes, with the following conditions: collision gas, He; collision energy amplitude, 1.3 V; nebulizer and drying gas, N_2_, 7 L/min; pressure of nebulizer gas, 30 psi; dry temperature, 340°C; flow rate, 1.0 mL/min; solvent split ratio 1 : 9; scan range,* m/z* 100–1000.

### 2.8. Identification of Propolis Constituents

**18** and** 22 **were directly identified in the DCM extract by HPLC/UV/MS and comparison with the literature data [31, 32], whereas** 3**,** 6**,** 7**,** 8**,** 10**, and** 32** were compared with authentic standards (Sigma-Aldrich and Acros organics, cf.  Section 2.1). A flash chromatography was then carried out in order to identify the other phenolic constituents. 50.0 g of pulverized propolis was firstly extracted with cyclohexane (3 × 200 mL, 2 h, room temperature) to eliminate waxes. After filtration, the residue was extracted with DCM (5 × 200 mL, 2 h, room temperature) to give 25.0 g of dry DCM extract (50% yield). 21.0 g of this extract was fractionated using a CombiFlash Teledyne ISCO apparatus and a prepacked silica gel column (Interchim PF-50SI HC/300 g, 50 *μ*m), at a flow rate of 100 mL/min and with the following gradient elution system: cyclohexane (C_6_H_12_) 100% (2.0 L), C_6_H_12_ : EtOAc 90 : 10 (1.7 L), C_6_H_12_ : EtOAc 90 : 10 to 80 : 20 (2.2 L), 80 : 20 to 70 : 30 (2.5 L), C_6_H_12_ : EtOAc 70 : 30 to 60 : 40 (2.2 L), and C_6_H_12_ : EtOAc 60 : 40 to 50 : 50 (3.0 L) then DCM : MeOH 96 : 4 (2.2 L). UV detection (*λ* 254 and 280 nm) and TLC monitoring allowed collecting 21 fractions (*F1–21*).** 48** [33] was identified in* F1*,** 14** [34] and** 17** [31] were identified in* F11*,** 1** and** 2** [19] were identified in* F13*, and finally** 23** [31] was identified in* F15* by HPLC/UV/MS and comparison with the literature data. The remaining constituents were isolated and identified through 1D and 2D NMR analysis (cf.  Section 2.2). 200 mg of* F1* was chromatographed on a silica gel column (Grace, 24 g) by flash chromatography at a flow rate of 25 mL/min with a mixture of C_6_H_12_ and EtOAc (B) [gradient: 1% B (30 min), 2% B (5 min), 2–5% B (2 min), 5% B (2 min), 5–30% B (1 min), and 30% B (5 min)] to give** 46** [33, 35] (5 mg),** 47** [33, 36] (3 mg), and** 49** [37] (5 mg).* F2* (1.5 g) gave** 43** [38] whereas* F4* (696 mg) and* F5* (384 mg) allowed us to, respectively, identify** 44** [37, 39] and** 45** [40]. 500 mg of* F6* (1.6 g) was chromatographed on reverse-phase- (RP-) Flash chromatography (Interchim column PF-30C18 HC/6 g, 30 *μ*m) at a flow rate of 15 mL/min with water and MeOH (B) [gradient: 25–30% B (20 min), 30–40% B (2 min), 40% B (8 min), 40–45% B (1 min), 45% B (12 min), 45–50% B (2 min), and 50% B (20 min)] to give** 9** [31] (10 mg),** 25** [38] (126 mg), and a mixture of** 35 **and** 36** [34] (15 mg). Similarly 500 mg of* F7* was fractionated [gradient: 30–50% B (25 min), 50–60% B (25 min), and 60–65% B (20 min)] to give** 11** [41] (2 mg),** 33** [31, 42] (128 mg),** 34** [38] (65 mg), and** 42** [31] (41 mg) whilst 500 mg of* F8* [gradient: 30–45% B (20 min), 45% B (30 min), 45–48% B (5 min), 48–55% B (5 min), 55% B (8 min), 55–60% B (1 min), and 60% B (12 min)] gave** 13** [38] (3 mg),** 28** [31, 43] (224 mg), and** 41** [34] (28 mg). 500 mg of* F9* [gradient: 30–60% B (50 min) and 60–65% B (5 min)] yielded mixture of** 4** [34] and** 5** [44, 45] (8 mg),** 15** [43] (33 mg),** 24** [32, 46] (16 mg),** 32** [38] (150 mg), and another mixture of** 37** [16] and** 38** [32] (3 mg).** 27** [34] and** 29** [31] were identified from* F10* (1.3 g). 500 mg of* F11* [flow rate of 20 mL/min, gradient: 25–75% B (55 min)] gave** 26** [47] (82 mg),** 31** [31] (56 mg), and** 39** [32] (70 mg). 300 mg of* F13* [flow rate of 15 mL/min, gradient: 30–45% B (20 min), 45% B (20 min), 45–50% B (10 min), 50–60% B (5 min), and 60% B (5 min)] gave** 10 **[31] (2 mg) and** 16** [38] (3 mg).** 30 **[48] was directly identified in* F17* (543 mg). 500 mg of* F18* [gradient: 40% B (7 min), 40–50% B (2 min), and 50% B (30 min)] gave a mixture of** 8** and** 12** (cf*. F19*) together with the new compound** 40** (12 mg). 500 mg of* F19 *was chromatographed [gradient: 25% B (25 min), 25–35% B (1 min), and 35% B (20 min)] to give** 8** (8 mg) and** 12** (26 mg) [31]. Finally 500 mg of* F20 *allowed us to isolate [gradient: 30–40% B (20 min), 40–43% B (20 min), and 43–50% B (10 min)]** 19** [32] (3 mg) and a mixture of** 20** [49] and** 21** [50] (7 mg).

## 3. Results and Discussion

### 3.1. Antifungal and Antibacterial Activities

Table 1 shows the minimum inhibitory concentration of at least 80% of fungal growth (MIC_80_) obtained with E1–6 extracts for* Candida albicans*,* C. glabrata*, and* Aspergillus fumigatus*. E1 did not exhibit any interesting antifungal activity (MIC_80_ > 250 *μ*g/mL for the three strains) whereas E2–6 showed significant antifungal activities (MIC_80_ between 16 and 31 *μ*g/mL) on both* C. albicans* and* C. glabrata*. These results are in agreement with those previously obtained for an Argentinian propolis on several* Candida* species (MIC_100_ in a range of 31 to 125 *μ*g/mL) [51] as well as with Greece and Cyprus ones (MIC_100_ 20 *μ*g/mL) [21]. E2–6 also exhibited a weak activity towards* A. fumigatus* (MIC_80_ 250 *μ*g/mL).

According to Ríos and Recio [52] a MIC_100_ < 100 *μ*g/mL should be considered as a promising value for a crude extract (versus 10 *μ*g/mL for pure compounds). This is the reason why Table 2 gives the results of the antibacterial activity of E1–6 at the concentration of 100 *μ*g/mL for 28 strains of Gram-negative and Gram-positive bacteria.

Results showed that Gram-negative bacteria were not susceptible to E1–6 at this concentration. In contrast, organic solvents extracts were active on several Gram-positive bacteria such as* Corynebacterium striatum* (sometimes involved in pleuropulmonary infections) (E2–5) and especially* Staphylococcus aureus,* including for the latter several methicillin-resistant (MRSA) and methicillin-susceptible (MSSA) clinical isolates (E5-6). Sometimes called “golden staph,”* S. aureus* is the most pathogenic species of* Staphylococcus* genus. It might cause food poisoning, skin infections, abscesses, and diseases like pneumonia, meningitis, and sepsis.* S. aureus* is additionally one of the major causes of hospital-acquired infections, and the treatment of some multiresistant strains has become quite problematic. Among them, MRSA appears in France as one of the most commonly multiresistant strains encountered in hospitals.

MIC_100_ of E1–6 were determined on the 6 susceptible Gram-positive strains as well as on 8 other MRSA and MSSA strains. Results are given in Table 3.

E1 did not show any interesting activity on the 14 studied strains (MIC_100_ > 100 *μ*g/mL). E2–6 showed interesting activities against* Corynebacterium striatum* with MIC_100_ ranging from 63 to 90 *μ*g/mL. E5 and E6 exhibited the best antibacterial activities against the* Staphylococcus *strains with MIC_100_ up to 57 and 30 *μ*g/mL, respectively. Among the alcoholic extracts, only E4 showed a moderate activity (MIC_100_ 90 *μ*g/mL) against* S. aureus* and one MRSA whereas E2 and E3 appeared as inactive. These overall activities therefore appeared to be better than those reported by Grange and Davey for the antibacterial activity of a French propolis on* S. aureus* and MRSA (MIC_100_ 188–375 *μ*g/mL) [23]. Our global antibacterial activity against MRSA and MSSA could be compared with those reported for propolis collected in Solomon Islands, exhibiting MIC_100_ between 64 and 128 *μ*g/mL [6]. Similarly E4 was more active than a methanolic propolis extract from Jordan (585 *μ*g/mL against* S. aureus* and 4700 *μ*g/mL against MRSA) [20].

These results suggested that antifungal and antibacterial activities of propolis extracts could be related to their flavonoids contents [24]. Indeed, whereas E1–6 exhibited high total polyphenol contents (239–281 mg GAE/g), only those showing both high flavone/flavonol (FF) and flavanone/dihydroflavonol (FD) contents (i.e., E5-6) were active on the studied strains. In addition the higher the cumulative contents FF+FD were, the stronger the antibacterial activity was, as shown with E5 (254 mg/g) and E6 (236 mg/g) > E2–4 (220–228 mg/g). These results are in agreement with those reported by Velazquez et al. [15] for different Mexican propolis collected in Sonora State where EEP from the areas of Ures (410 mg/g), Caborca (332 mg/g), and Pueblo de Alamos (209 mg/g) showed MIC_100_ against* S. aureus* of 100, 200, and >400 *μ*g/mL, respectively.

### 3.2. Chemical Composition

**Figure 1:**
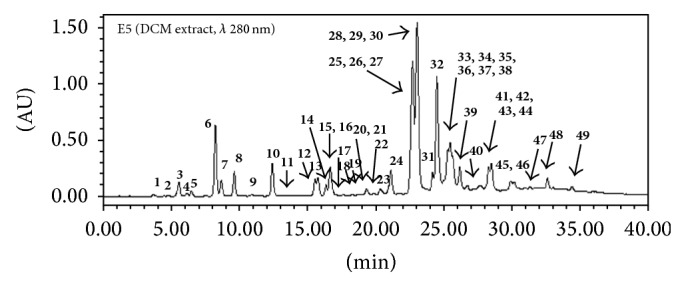
HPLC chromatograms of E5:** 1** 3,4-dihydroxybenzaldehyde,** 2** 4-hydroxybenzoic acid,** 3** caffeic acid,** 4** vanillin,** 5** 4-hydroxyacetophenone,** 6** *p*-coumaric acid,** 7** ferulic acid,** 8** isoferulic acid,** 9** benzoic acid,** 10** 3,4-dimethoxycinnamic acid,** 11** 3-phenylpropanoic acid,** 12** pinobanksin-5-methyl ether,** 13** cinnamic acid,** 14** 4-methoxycinnamic acid,** 15** pinobanksin,** 16** naringenin,** 17** quercetin,** 18** quercetin-3-methyl ether,** 19** pinocembrin-5-methyl ether,** 20** 1,3-di-*p*-coumaroylglycerol,** 21** 1-*p*-coumaroyl-3-feruloylglycerol,** 22** kaempferol,** 23** apigenin,** 24** cinnamylidene acetic acid,** 25** pinocembrin,** 26** benzyl caffeate,** 27** isopent-3-enyl caffeate,** 28** pinobanksin-3-acetate,** 29** prenyl caffeate,** 30** 2-acetyl-1,3-dicoumaroylglycerol,** 31** phenylethyl caffeate (CAPE),** 32** chrysin,** 33** benzyl* p*-coumarate,** 34** galangin,** 35** benzyl ferulate,** 36** prenyl ferulate,** 37** kaempferide,** 38** rhamnocitrin,** 39** cinnamyl caffeate,** 40** 8-[(E)-phenylprop-2-en-1-one]-5-methoxy-(±)-catechin (new),** 41** cinnamyl isoferulate,** 42** cinnamyl* p*-coumarate,** 43** pinostrobin,** 44** alpinone-3-acetate,** 45** tectochrysin,** 46** benzyl cinnamate,** 47** cinnamyl benzoate,** 48** cinnamyl cinnamate, and** 49** cinnamyl cinnamylidene acetate.

Figure 1 shows the HPLC chromatogram of the DCM extract E5. 48 compounds were identified by comparison with the literature data (UV/MS) and pure standards or, when needed, through ^1^H and ^13^C (1D and 2D) NMR analysis after compound isolation.

**Figure 2:**
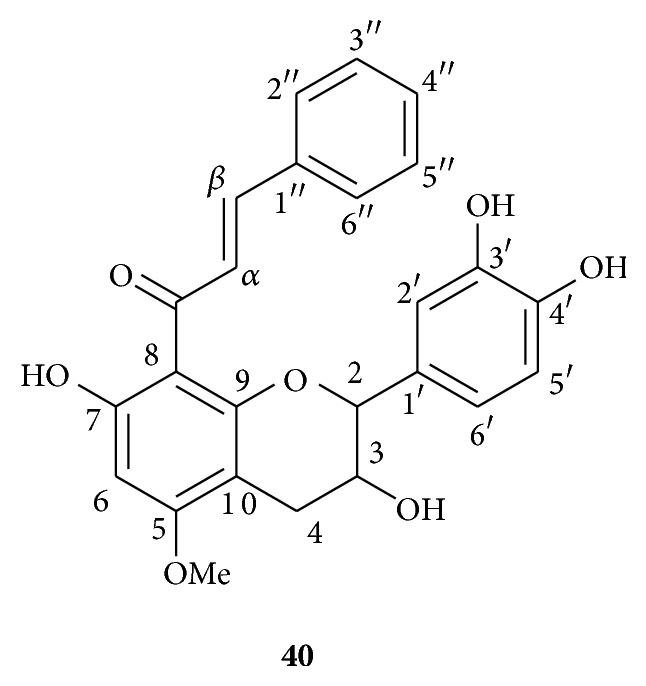
Chemical structure of the new compound** 40**.

Additionally a new flavan-3-ol was identified as the 8-[(E)-phenylprop-2-en-1-one]-5-methoxy-(±)-catechin** 40** (Figure 2).

**Figure 3:**
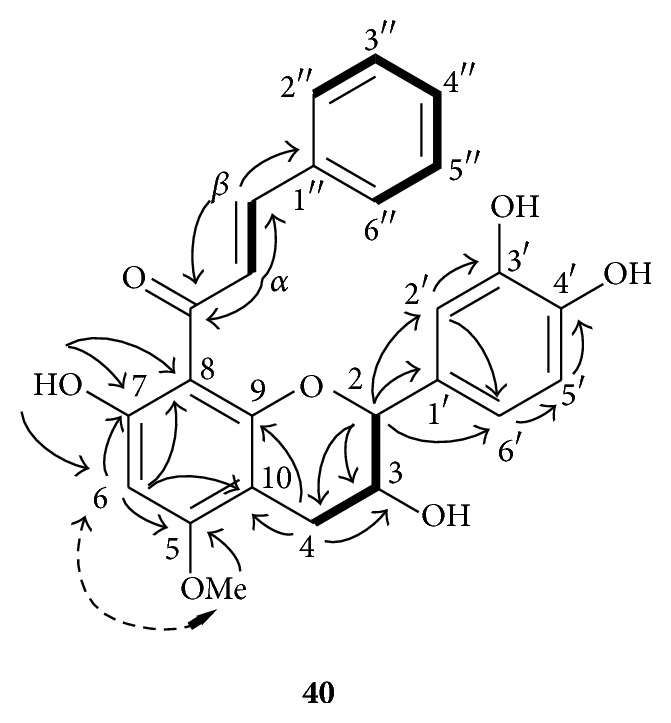
2D NMR studies of compound** 40**: COSY (bold lines), selected HMBC (solid arrows: ^1^H → ^13^C), and NOESY (dashed arrows) correlations.

Compound** 40** was obtained as a yellow amorphous solid (0.6 *μ*g/g of DCM extract). The molecular formula was determined as C_25_H_22_O_7_ by HRESIMS (found for [M+H]^+^ 435.1436; calculated 435.1438). The UV spectrum showed an absorption maximum at 350 nm. The IR spectrum indicated the presence of OH (3400 cm^−1^) as well as conjugated ketone carbonyl (1610 cm^−1^) groups. The ^1^H NMR spectrum exhibited signals due to a hydrogen-bonded OH at *δ*_H_ 14.49, two* trans*-olefinic protons (*δ*_H_ 8.06 and 7.63, 2 d, *J* = 15.7 Hz), aromatic rings (9H, *δ*_H_ 6.15–7.30), and one methoxyle (*δ*_H_ 3.92). It also showed the characteristic signals of a flavan-3-ol moiety at *δ*_H_ 4.68 (1H, d, *J* = 8.9 Hz, H_2_), 4.21 (1H, m, H_3_), 3.07 (1H, dd, *J* = 16.2, 5.7 Hz, H_4a_), and 2.53 (1H, dd, *J* = 16.2, 9.5 Hz, H_4b_). The ^13^C NMR and HMQC spectra confirmed the presence of 25 carbons with typical flavan-3-ol signals at *δ*_C_ 84.2 (C_2_), 66.8 (C_3_), and 30.6 (C_4_). In the ^1^H NMR spectrum, the signals at *δ*_H_ 7.11 (1H, d, *J* = 1.4 Hz), 6.96 (1H, dd, *J* = 8.4, 1.4 Hz), and 6.91 (1H, d, *J* = 8.4 Hz) suggested the presence of a 1′,3′,4′-trisubstituted ring B whereas a singlet at *δ*_H_ 6.15 (1H) indicated a pentasubstituted ring A. Two multiplets at *δ*_H_ 7.17 (2H) and 7.29 (3H) revealed the presence of a phenyl residue. The HMBC spectrum showed a long-range correlation between the two* trans*-olefinic protons [*δ*_H_ 7.63 (1H, d, *J* = 15.7, H_*α*_) and 8.06 (1H, d, *J* = 15.7, H_*β*_)] and the ketone carbon at *δ*_C_ 193.2. This correlation revealed the presence of an *α*,*β*-unsaturated ketone group. The* trans*-olefinic proton H_*β*_ at *δ*_H_ 8.06 was also correlated with the phenyl quaternary carbon at *δ*_C_ 136.2 (C_1′′_). This correlation implied the presence of a (*2E*)-4-phenylprop-2-en-1-one moiety. A correlation between the methoxyle protons (*δ*_H_ 3.92) and the carbon at *δ*_C_ 165.1 (C_5_) proved that the OCH_3_ was attached to C_5_. The NOESY spectrum showed that this methoxyle was spatially close to the proton at *δ*_H_ 6.15 (H_6_), whereas a long-range COSY indicated a correlation between H_6_ and one of the hydroxyl groups at *δ*_H_ 14.49 (OH_7_). Therefore a (*2E*)-4-phenylprop-2-en-1-one moiety was located at C_8_ (*δ*_C_ 105.9). Finally, it appeared that the aromatic ring B was substituted at C_3′_ and C_4′_ by two hydroxyl groups (NMR spectra cf. supporting information 2). ^1^H and ^13^C NMR data together with 2D NMR correlations for** 40** are summarized in Table 4 and Figure 3.

**Figure 4:**
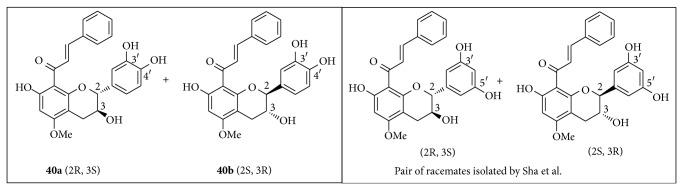
Structures of 8-[(E)-phenylprop-2-en-1-one]-(2R,3S)-5-methoxycatechin (**40a**) and 8-[(E)-phenylprop-2-en-1-one]-(2S,3R)-5-methoxycatechin (**40b**) and a similar pair of racemates isolated by Sha et al. 2009 [26].

**40** had no optical rotation and, thus, was isolated here as a racemate mixture of 8-[(E)-phenylprop-2-en-1-one]-(2R,3S)-5-methoxycatechin (**40a**) and 8-[(E)-phenylprop-2-en-1-one]-(2S,3R)-5-methoxycatechin (**40b**). Sha et al. already isolated a similar compound, only differing from** 40** by a 1′,3′,5′-trisubstituted aromatic ring B, from a Chinese propolis [26] (Figure 4).

### 3.3. Antifungal and Antibacterial Activities of **40**

The new flavan-3-ol** 40** did not show any antifungal activity on the three strains studied (Table 5). However, though active neither on* S. aureus* nor on MSSA, its MIC_100_ on MRSA numbers 23 and 24 were lower or equal to 10 *μ*g/mL (close to oxacillin: ≥4 *μ*g/mL).

### 3.4. Major Compounds Activities

Antifungal and antibacterial activities were then individually evaluated for the five major compounds identified in E2–6, namely, pinobanksin-3-acetate (**28**), pinocembrin (**25**), chrysin** (32**), galangin (**34**), and prenyl caffeate (**29**) [24]. Their MIC_80_ towards* C. albicans*,* C. glabrata*, and* A. fumigatus* as well as their MIC_100_ towards* S. aureus*, MRSA, and MSSA are given in Table 6.

Pinobanksin-3-acetate (**28**), chrysin (**32**), and galangin (**34**) appeared as inactive. Pinocembrin (**25**) showed a moderate activity towards* Candida albicans*,* C. glabrata* (MIC_80_ 62–125 *μ*g/mL), and* S. aureus* (MIC_100_ 100 *μ*g/mL). Overall prenyl caffeate (**29**) exhibited the best activities (MIC_100_ up to 16 *μ*g/mL against* C. glabrata* and up to 63 *μ*g/mL against* S. aureus* and MRSA). Even so it appeared that these compounds were not individually as active as it could be expected from E5-6 results (MIC_100_ 30–97 *μ*g/mL). As far as* S. aureus* and MRSA are concerned, this kind of synergistic effects was recently pointed out by Darwish et al. [20] who evaluated the antibacterial activities of pinobanksin-3-acetate, pinocembrin, and chrysin isolated from a Jordanian propolis. Therefore these results are also in agreement with Kujumgiev et al. stating that, in spite of a great chemodiversity, no specific compounds can be associated with the antimicrobial activities of propolis extracts whereas, obviously, different flavonoid combinations are essential for these activities [7]. The antimicrobial of propolis extracts most probably involves a complex mechanism. It can be attributed to the synergistic effects of phenolic compounds such as cinnamic acid and ester derivatives, including caffeic acid and CAPE, as well as flavonoids including quercetin and naringenin [17, 53, 54]. Indeed, each of these compounds would be able to increase membrane permeability and inhibit bacterial mobility [54], thus contributing to the antimicrobial activity of propolis but also to its synergism with other antibiotics [53, 55, 56]. It is the reason why Stepanović et al. could notice the antibacterial and synergistic actions of propolis extracts with ampicillin, ceftriaxone, and doxycycline towards* Staphylococcus aureus* and with nystatin towards* Candida albicans*, stating that the bacterial resistance to antibiotics had no influence on the susceptibility to propolis extracts [57].* In vitro* studies of synergism carried by Fernandes Jr. et al. also revealed synergistic effects of EEP with chloramphenicol, gentamicin, netilmicin, tetracycline, vancomycin, and clindamycin [58]. Therefore our findings are in total accordance with these results and, now that antibiotic resistance to bacteria has become a major public health concern [59], could bring valuable knowledge to develop new antimicrobial drugs for challenging* S. aureus* infections.

## 4. Conclusions

On the basis of these results, it may be concluded that organic solvents extracts of a French poplar type propolis are associated with a good antifungal activity towards* Candida albicans* and* C. glabrata*, correlated with high flavonoid contents. However only DCM based extracts (E5-6) showed a significant antibacterial activity against both methicillin-resistant and methicillin-susceptible* Staphylococcus aureus* strains. Unfortunately these extracts are not compatible with a pharmaceutical use because of their toxicity, whereas EtOH based extracts were not as active as expected. Therefore it would be interesting to develop some alternative extraction of propolis using a nontoxic solvent such as subcritical water. In addition, it should be noticed that, as an intrinsic polytherapy, propolis may also circumvent the development of drug resistance by bacteria [60].

## Supplementary Material

The collection sites of the different propolis samples as well as the full NMR data set of new compound 40 (8-[(E)-phenylprop-2-en-1-one]-5-methoxy-(±)-catechin) are available online as Supplementary Material.

## Figures and Tables

**Table 1:** Antifungal activity against *Candida albicans*, *C. glabrata,* and *Aspergillus fumigatus*.

Extract	Solvent	*C. albicans *	*C. glabrata *	*A. fumigatus *
		MIC_80_ (*µ*g/mL)		
E1	H_2_O	>250	>250	>250
E2	95% EtOH	31.25	15.63	250
E3	70% EtOH	31.25	31.25	250
E4	MeOH	31.25	31.25	250
E5	DCM	31.25	31.25	250
E6	Mixed solvents	15.63	31.25	250
Amphotericin B		0.125	0.125	6

**Table 2:** Antibacterial activity of E1–6 against 28 Gram-negative and Gram-positive strains.

Number	Bacterial strains	Extracts (100 *µ*g/mL)					
		E1: H_2_O	E2: 95% EtOH	E3: 70% EtOH	E4: MeOH	E5: DCM	E6: mixed solvents
*Gram-negative: *							
1	*Acinetobacter baumannii* (RCH)	−	−	−	−	−	−
2	*Acinetobacter baumannii* (SAN008)	−	−	−	−	−	−
3	*Acinetobacter baumannii* (12)	−	−	−	−	−	−
4	*Acinetobacter baumannii* (AYE)	−	−	−	−	−	−
5	*Acinetobacter baumannii *(CIP7034)	−	−	−	−	−	−
6	*Acinetobacter baumannii* (CIP107292)	−	−	−	−	−	−
7	*Acinetobacter baumannii* (CIP5377)	−	−	−	−	−	−
8	*Enterobacter cloacae* (0705A1743)	−	−	−	−	−	−
9	*Enterobacter aerogenes* (0705A0867)	−	−	−	−	−	−
10	*Escherichia coli* (ATCC25922)	−	−	−	−	−	−
11	*Escherichia coli* (0705A0434)	−	−	−	−	−	−
12	*Klebsiella oxytoca* (0705C0187)	−	−	−	−	−	−
13	*Pseudomonas aeruginosa* (ATCC27853)	−	−	−	−	−	−
14	*Pseudomonas aeruginosa *(0704C0134)	−	−	−	−	−	−
15	*Pseudomonas aeruginosa *(0703C0259)	−	−	−	−	−	−
16	*Salmonella enteritidis* (4)	−	−	−	−	−	−

*Gram-positive: *							
17	*Corynebacterium striatum (56) *	−	+	+	+	+	+
18	*Enterococcus faecalis *(11003508001)	−	−	−	−	−	−
19	*Enterococcus faecalis *(11003492701)	−	−	−	−	−	−
20	*Enterococcus faecalis *(11004774001)	−	−	−	−	−	−
21	*Enterococcus faecium *(11502441401)	−	−	−	−	−	−
22	*Staphylococcus aureus* (ATCC25923)	−	−	−	+	+	+
23	*MRSA *(0706C0025)	−	−	−	+	+	+
24	*MRSA* (0702E0196)	−	−	−	−	+	+
25	*MSSA* (0703H0036)	−	−	−	−	−	−
26	*MSSA* (0701A0095)	−	−	−	−	+	+
27	*S. epidermidis methiS* ^a^ * *(12004523201)	−	−	−	−	−	−
28	*S. epidermidis methiR* ^b^ * *(12552599902)	−	−	−	−	−	−

−: no antibacterial activity, +: antibacterial activity, ^a^ *methicillin-Susceptible*, ^b^ *methicillin-Resistant*.

**Table 3:** MICs of E1–6 against 14 Gram-positive strains including MRSA and MSSA.

Number	Bacterial strains	MIC_100_ (*µ*g/mL)						
		E1: H_2_O	E2: 95% EtOH	E3: 70% EtOH	E4: MeOH	E5: DCM	E6: mixed solvents	Oxacillin
17	*Corynebacterium striatum *	>100	83 ± 6	90 ± 0	77 ± 12	63 ± 15	87 ± 21	—
22	*Staphylococcus aureus* (ATCC25923)	>100	>100	>100	90 ± 0	60 ± 10	67 ± 15	≤0.25
23	*MRSA *(0706C0025)	>100	>100	>100	90 ± 0	57 ± 12	30 ± 0	≥4
24	*MRSA* (0702E0196)	>100	>100	>100	>100	80 ± 10	77 ± 23	≥4
25	*MSSA* (0703H0036)	>100	>100	>100	>100	>100	>100	≤0.25
26	*MSSA* (0701A0095)	>100	>100	>100	>100	87 ± 6	83 ± 29	≤0.25
29	*MRSA *(11004533801)	>100	>100	>100	>100	80 ± 0	87 ± 21	≥4
30	*MRSA *(11004691801)	>100	>100	>100	>100	77 ± 6	67 ± 23	≥4
31	*MRSA *(11004787401)	>100	>100	>100	>100	97 ± 6	>100	≥4
32	*MRSA *(11006153901)	>100	>100	>100	>100	77 ± 6	73 ± 29	≥4
33	*MSSA *(11004327701)	>100	>100	>100	>100	77 ± 6	73 ± 12	0.25
34	*MSSA *(11004480701)	>100	>100	>100	>100	80 ± 0	97 ± 12	0.5
35	*MSSA *(11004691801)	>100	>100	>100	>100	77 ± 6	90 ± 17	0.5
36	*MSSA* (11004010401)	>100	>100	>100	>100	77 ± 6	90 ± 0	≤0.25

**Table 4:** ^1^H and ^13^C NMR data of the new compound **40** (in acetone-*d*6).

Position	**40**	
	*δ*_H_, mult. (*J* in Hz)	*δ*_C_, mult.
2	4.68, d (8.9)	84.2, CH
3	4.21, m	66.8, CH
4	a 3.07, dd (16.2, 5.7)	30.6, CH_2_
	b 2.53, dd (16.2, 9.5)	
5		165.1, qC
6	6.15, s	93.5, CH
7		168.1, qC
8		105.9, qC
9		157.6, qC
10		102.4, qC
1′		130.7, qC
2′	7.11, d (1.4)	116.2, CH
3′		146.6, qC
4′		146.2, qC
5′	6.91, d (8.4)	116.0, CH
6′	6.96, dd (8.4, 1.4)	121.3, CH
1′′		136.2, qC
2′′	7.28, m	129.2, CH
3′′	7.16, m	129.7, CH
4′′	7.28, m	130.8, CH
5′′	7.16, m	129.7, CH
6′′	7.28, m	129.2, CH
OH-7	14.49, s	
*α*	7.63, d (15.7)	143.1, CH
*β*	8,06, d (15.7)	128.4, CH
C=O		193.2, qC
OCH_3_-5	3.92, s	56.5, CH_3_

**Table 5:** Antifungal and antibacterial activities of **40**.

Antifungal activity		Antibacterial activity		
Fungal strains	MIC_80_ (*µ*g/mL)	Number	Bacterial strains	MIC_100_ (*µ*g/mL)
*C. albicans *	>250	22	*S. aureus* (ATCC25923)	>100
*C. glabrata *	>250	23	*MRSA *(0706C0025)	≤10
*A. fumigatus *	>250	24	*MRSA * (0702E0196)	≤10
		25	*MSSA * (0703H0036)	>100
		26	*MSSA * (0701A0095)	>100

**Table 6:** Antifungal and antibacterial activities of five major compounds.

	Antifungal strains	MIC_80_ (*µ*g/mL)					
		Pinobanksin-3-acetate (**28**)	Pinocembrin (**25**)	Chrysin (**32**)	Galangin (**34**)	Prenyl caffeate (**29**)	Amphotericin B
	*Candida albicans *	250	62	>250	>250	62	0.125
	*Candida glabrata *	250	125	>250	>250	16	0.125
	*Aspergillus fumigatus *	>250	250	>250	>250	125	6

Number	Bacterial strains	MIC_100_ (*µ*g/mL)					
		Pinobanksin-3-acetate (**28**)	Pinocembrin (**25**)	Chrysin (**32**)	Galangin (**34**)	Prenyl caffeate (**29**)	Oxacillin

22	*Staphylococcus aureus* (ATCC25923)	>100	100 ± 0	>100	>100	63 ± 6	≤0.25
23	*MRSA *(0706C0025)	>100	>100	>100	>100	70 ± 0	≥4
24	*MRSA * (0702E0196)	>100	>100	>100	>100	70 ± 0	≥4
25	*MSSA * (0703H0036)	>100	>100	>100	>100	93 ± 6	≤0.25
26	*MSSA * (0701A0095)	>100	>100	>100	>100	93 ± 6	≤0.25

## Abbreviations

APCI: Atmospheric pressure chemical ionization
ATCC: American Type Culture Collection
AYE: Patient code
CAPE: Caffeic acid phenylethyl ester
CBS: Centraal Bureau voor Schimmelcultures
CIP: Collection de l'Institut Pasteur
COSY: Correlation spectroscopy
DCM: Dichloromethane
DMSO: Dimethyl sulfoxide
EEP: Ethanolic extract of propolis
ESI: Electrospray ionization
FD: Flavanone/dihydroflavonol
FF: Flavone/flavonol
GAE: Gallic acid equivalent
HMBC: Heteronuclear multiple bond correlation
HMQC: Heteronuclear multiple quantum correlation
HPLC: High performance liquid chromatography
HRESIMS: High resolution electrospray ionization mass spectrometry
IR: Infrared
LMA: Laboratoire de Mycologie d'Angers
MIC: Minimum inhibitory concentration
MRSA: Methicillin-resistant* Staphylococcus aureus*
MS: Mass spectrometry
MSSA: Methicillin-susceptible* Staphylococcus aureus*
NCCLS: National Committee for Clinical Laboratory Standards
NMR: Nuclear magnetic resonance
NOESY: Nuclear Overhauser effect spectroscopy
RCH: Patient code
RP: Reversed phase
RPMI: Roswell Park Memorial Institute
UV: Ultraviolet
YPDA: Yeast peptone dextrose agar.

## References

[1] Cottica S. M., Sawaya A. C. H. F., Eberlin M. N., Franco S. L., Zeoula L. M., Visentainer J. V. (2011). Antioxidant activity and composition of propolis obtained by different methods of extraction. *Journal of the Brazilian Chemical Society*.

[2] Miguel M. G., Nunes S., Dandlen S. A., Cavaco A. M., Antunes M. D. (2010). Phenols and antioxidant activity of hydro-alcoholic extracts of propolis from Algarve, South of Portugal. *Food and Chemical Toxicology*.

[3] Gülçin I., Bursal E., Şehitoĝlu M. H., Bilsel M., Gören A. C. (2010). Polyphenol contents and antioxidant activity of lyophilized aqueous extract of propolis from Erzurum, Turkey. *Food and Chemical Toxicology*.

[4] Ota C., Unterkircher C., Fantinato V., Shimizu M. T. (2001). Antifungal activity of propolis on different species of *Candida*. *Mycoses*.

[5] Sawaya A. C. H. F., Palma A. M., Caetano F. M. (2002). Comparative study of in vitro methods used to analyse the activity of propolis extracts with different compositions against species of Candida. *Letters in Applied Microbiology*.

[6] Raghukumar R., Vali L., Watson D., Fearnley J., Seidel V. (2010). Antimethicillin-resistant *Staphylococcus aureus* (MRSA) activity of ‘pacific propolis’ and isolated prenylflavanones. *Phytotherapy Research*.

[7] Kujumgiev A., Tsvetkova I., Serkedjieva Y., Bankova V., Christov R., Popov S. (1999). Antibacterial, antifungal and antiviral activity of propolis of different geographic origin. *Journal of Ethnopharmacology*.

[8] Popova M., Silici S., Kaftanoglu O., Bankova V. (2005). Antibacterial activity of Turkish propolis and its qualitative and quantitative chemical composition. *Phytomedicine*.

[9] Castaldo S., Capasso F. (2002). Propolis, an old remedy used in modern medicine. *Fitoterapia*.

[10] Marcucci M. (1995). Propolis: chemical composition, biological properties and therapeutic activity. *Apidologie*.

[11] Bankova V. S., De Castro S. L., Marcucci M. C. (2000). Propolis: recent advances in chemistry and plant origin. *Apidologie*.

[12] Kumazawa S., Hamasaka T., Nakayama T. (2004). Antioxidant activity of propolis of various geographic origins. *Food Chemistry*.

[13] Bankova V. (2005). Chemical diversity of propolis and the problem of standardization. *Journal of Ethnopharmacology*.

[14] Salatino A., Fernandes-Silva C. C., Righi A. A., Salatino M. L. F. (2011). Propolis research and the chemistry of plant products. *Natural Product Reports*.

[15] Velazquez C., Navarro M., Acosta A. (2007). Antibacterial and free-radical scavenging activities of Sonoran propolis. *Journal of Applied Microbiology*.

[16] Agüero M. B., Gonzalez M., Lima B. (2010). Argentinean propolis from *Zuccagnia punctata* cav. (Caesalpinieae) exudates: phytochemical characterization and antifungal activity. *Journal of Agricultural and Food Chemistry*.

[17] Santos F. A., Bastos E. M. A., Uzeda M. (2002). Antibacterial activity of Brazilian propolis and fractions against oral anaerobic bacteria. *Journal of Ethnopharmacology*.

[18] Koo H., Gomes B. P. F. A., Rosalen P. L., Ambrosano G. M. B., Park Y. K., Cury J. A. (2000). In vitro antimicrobial activity of propolis and Arnica montana against oral pathogens. *Archives of Oral Biology*.

[19] Mohammadzadeh S., Shariatpanahi M., Hamedi M., Ahmadkhaniha R., Samadi N., Ostad S. N. (2007). Chemical composition, oral toxicity and antimicrobial activity of Iranian propolis. *Food Chemistry*.

[20] Darwish R. M., Fares R. J. A., Zarga M. H. A., Nazer I. K. (2010). Antibacterial effect of Jordanian propolis and isolated flavonoids against human pathogenic bacteria. *African Journal of Biotechnology*.

[21] Kalogeropoulos N., Konteles S. J., Troullidou E., Mourtzinos I., Karathanos V. T. (2009). Chemical composition, antioxidant activity and antimicrobial properties of propolis extracts from Greece and Cyprus. *Food Chemistry*.

[22] Hegazi A. G., Abd El Hady F. K., Abd Allah F. A. M. (2000). Chemical composition and antimicrobial activity of European propolis. *Zeitschrift fur Naturforschung, Section C*.

[23] Grange J. M., Davey R. W. (1990). Antibacterial properties of propolis (bee glue). *Journal of the Royal Society of Medicine*.

[24] Boisard S., Le Ray A.-M., Gatto J. (2014). Chemical composition, antioxidant and anti-AGEs activities of a French poplar type propolis. *Journal of Agricultural and Food Chemistry*.

[25] de Castro Ishida V. F., Negri G., Salatino A., Bandeira M. F. C. L. (2011). A new type of Brazilian propolis: prenylated benzophenones in propolis from Amazon and effects against cariogenic bacteria. *Food Chemistry*.

[26] Sha N., Guan S.-H., Lu Z.-Q. (2009). Cytotoxic constituents of Chinese propolis. *Journal of Natural Products*.

[27] Alomar K., Gaumet V., Allain M., Bouet G., Landreau A. (2012). Synthesis, crystal structure, characterisation, and antifungal activity of 3-thiophene aldehyde semicarbazone (3STCH), 2,3-thiophene dicarboxaldehyde bis(semicarbazone) (2,3BSTCH_2_) and their nickel (II) complexes. *Journal of Inorganic Biochemistry*.

[28] National Commitee for Clinical Laboratory Standards (1997). *Reference Method for Broth Dilution Antifungal Susceptibility Testing of Yeasts*.

[29] National Commitee for Clinical Laboratory Standards (2002). Reference method for broth dilution antifungal susceptibility testing of filamentous fungi, approved standard. *NCCLS Document*.

[30] Alomar K., Landreau A., Kempf M., Khan M. A., Allain M., Bouet G. (2010). Synthesis, crystal structure, characterization of zinc(II), cadmium(II) complexes with 3-thiophene aldehyde thiosemicarbazone (3TTSCH). Biological activities of 3TTSCH and its complexes. *Journal of Inorganic Biochemistry*.

[31] Pellati F., Orlandini G., Pinetti D., Benvenuti S. (2011). HPLC-DAD and HPLC-ESI-MS/MS methods for metabolite profiling of propolis extracts. *Journal of Pharmaceutical and Biomedical Analysis*.

[32] Falcão S. I., Vale N., Gomes P. (2013). Phenolic profiling of Portuguese propolis by LC-MS spectrometry: uncommon propolis rich in flavonoid glycosides. *Phytochemical Analysis*.

[33] Li F., Awale S., Tezuka Y., Esumi H., Kadota S. (2010). Study on the constituents of mexican propolis and their cytotoxic activity against PANC-1 human pancreatic cancer cells. *Journal of Natural Products*.

[34] Rubiolo P., Casetta C., Cagliero C., Brevard H., Sgorbini B., Bicchi C. (2013). *Populus nigra* L. bud absolute: a case study for a strategy of analysis of natural complex substances. *Analytical and Bioanalytical Chemistry*.

[35] Sova M., Perdih A., Kotnik M. (2006). Flavonoids and cinnamic acid esters as inhibitors of fungal 17*β*-hydroxysteroid dehydrogenase: a synthesis, QSAR and modelling study. *Bioorganic & Medicinal Chemistry*.

[36] Correia R., DeShong P. (2001). Palladium-catalyzed arylation of allylic benzoates using hypervalent siloxane derivatives. *The Journal of Organic Chemistry*.

[37] Athikomkulchai S., Awale S., Ruangrungsi N., Ruchirawat S., Kadota S. (2013). Chemical constituents of Thai propolis. *Fitoterapia*.

[38] Bertelli D., Papotti G., Bortolotti L., Marcazzan G. L., Plessi M. (2012). 1H-NMR simultaneous identification of health-relevant compounds in propolis extracts. *Phytochemical Analysis*.

[39] Gripenberg J., Honkanen E., Silander K., Stenhagen E., Thorell B. (1956). The structure of alpinone. *Acta Chemica Scandinavica*.

[40] Park Y., Moon B.-H., Yang H., Lee Y., Lee E., Lim Y. (2007). Complete assignments of NMR data of 13 hydroxymethoxyflavones. *Magnetic Resonance in Chemistry*.

[41] Markham K. R., Mitchell K. A., Wilkins A. L., Daldy J. A., Lu Y. (1996). HPLC and GC-MS identification of the major organic constituents in New Zealand propolis. *Phytochemistry*.

[42] Bankova V. S. (1990). Synthesis of natural esters of substituted cinnamic acids. *Journal of Natural Products*.

[43] Fang J.-M., Su W.-C., Cheng Y.-S. (1988). Flavonoids and stilbenes from armand pine. *Phytochemistry*.

[44] Greenaway W., Scaysbrook T., Whatley F. R. (1988). Composition of propolis in Oxfordshire, U.K. and its relation to poplar bud exudate. *Zeitschrift für Naturforschung C: Journal of Biosciences*.

[45] Mazille F., Schoettl T., Lopez A., Pulgarin C. (2010). Physico-chemical properties and photo-reactivity relationship for para-substituted phenols in photo-assisted Fenton system. *Journal of Photochemistry and Photobiology A: Chemistry*.

[46] Sanyal R., Badami B. V. (2009). A new Synthesis of 3-arylpropenoic acids and 5-phenyl-2,4- pentadienoic acid from 4-acetyl-3-arylsydnones and arylaldehydes. *Organic Communications*.

[47] Yamauchi R., Kato K., Oida S., Kanaeda J., Ueno Y. (2014). Benzyl caffeate, an antioxidative compound isolated from propolis. *Bioscience, Biotechnology and Biochemistry*.

[48] Banskota A. H., Nagaoka T., Sumioka L. Y. (2002). Antiproliferative activity of the Netherlands propolis and its active principles in cancer cell lines. *Journal of Ethnopharmacology*.

[49] Koshino H., Terada S.-I., Yoshihara T. (1988). Three phenolic acid derivatives from stromata of *Epichloe typhina* on *Phleum pratense*. *Phytochemistry*.

[50] Cooper R., Gottlieb H. E., Lavie D. (1978). New phenolic diglycerides from Aegilops ovata. *Phytochemistry*.

[51] Agüero M. B., Svetaz L., Sánchez M. (2011). Argentinean Andean propolis associated with the medicinal plant *Larrea nitida* Cav. (Zygophyllaceae). HPLC–MS and GC–MS characterization and antifungal activity. *Food and Chemical Toxicology*.

[52] Ríos J. L., Recio M. C. (2005). Medicinal plants and antimicrobial activity. *Journal of Ethnopharmacology*.

[53] Krol W., Scheller S., Shani J., Pietsz G., Czuba Z. (1993). Synergistic effect of ethanolic extract of propolis and antibiotics on the growth of *Staphylococcus aureus*. *Arzneimittel-Forschung*.

[54] Mirzoeva O. K., Grishanin R. N., Calder P. C. (1997). Antimicrobial action of propolis and some of its components: the effects on growth, membrane potential and motility of bacteria. *Microbiological Research*.

[55] Bonvehí J. S., Coll F. V., Jordà R. E. (1994). The composition, active components and bacteriostatic activity of propolis in dietetics. *Journal of the American Oil Chemists' Society*.

[56] Wojtyczka R. D., Dziedzic A., Idzik D. (2013). Susceptibility of Staphylococcus aureus clinical isolates to propolis extract alone or in combination with antimicrobial drugs. *Molecules*.

[57] Stepanović S., Antić N., Dakić I., Švabić-Vlahović M. (2003). *In vitro* antimicrobial activity of propolis and synergism between propolis and antimicrobial drugs. *Microbiological Research*.

[58] Fernandes A., Balestrin E. C., Betoni J. E. C., Orsi R. D. O., da Cunha M. D. L. R. D. S., Montelli A. C. (2005). Propolis: anti-*Staphylococcus aureus* activity and synergism with antimicrobial drugs. *Memórias do Instituto Oswaldo Cruz*.

[59] Davies J., Davies D. (2010). Origins and evolution of antibiotic resistance. *Microbiology and Molecular Biology Reviews*.

[60] Pamplona-Zomenhan L. C., Pamplona B. C., da Silva C. B., Marcucci M. C., Mimica L. M. J. (2011). Evaluation of the *in vitro* antimicrobial activity of an ethanol extract of Brazilian classified propolis on strains of *Staphylococcus aureus*. *Brazilian Journal of Microbiology*.

