# Supplementary material for: Antifungal and Antibacterial Metabolites from a French Poplar Type Propolis
Source: Evid Based Complement Alternat Med. 2015 Mar 22;2015:319240. doi: 10.1155/2015/319240 (PMC4385655; doi:10.1155/2015/319240)
Supplement: Supplementary file 1 — The collection sites of the different propolis samples as well as the full NMR data set of new compound 40 (8-[(E)-phenylprop-2-en-1-one]-5-methoxy-(±)-catechin) are available online as Supplementary Material. [file 319240.f1.docx]

# Optional Supplementary Materials

**Additional file 1. Composition of the typical French batch**

Collection sites of the French propolis samples

**Additional file 2. NMR spectra of new compound 40**
